# Changes in Retinol Binding Protein 4 Level in Undernourished Children After a Nutrition Intervention Are Positively Associated With Mother’s Weight but Negatively With Mother’s Height, Intake of Whole Milk, and Markers of Systemic Inflammation: Results From a Community-Based Intervention Study

**DOI:** 10.1177/0379572120973908

**Published:** 2020-11-23

**Authors:** Subhasish Das, Md Amran Gazi, Md Mehedi Hasan, Shah Mohammad Fahim, Md Ashraful Alam, Md Shabab Hossain, Mustafa Mahfuz, Tahmeed Ahmed

**Affiliations:** 1 Nutrition and Clinical Services Division (NCSD), 56291 International Centre for Diarrhoeal Disease Research, Bangladesh (icddr, b), Dhaka, Bangladesh; 2 Department of Global Health, University of Washington, Seattle, WA, USA; 3 James P. Grant School of Public Health, BRAC University, Mohakhali, Dhaka 1212, Bangladesh

**Keywords:** retinol binding protein 4, childhood undernutrition, subcutaneous adiposity, maternal height, maternal weight, inflammation

## Abstract

**Background:**

The changes of plasma retinol binding protein 4 (RBP4) level after a nutrition intervention can indicate the metabolic changes associated with the delivered intervention.

**Objective:**

We investigated the changes in plasma RBP4 level among 12- to 18-month-old children after a nutrition intervention and measured its association with subcutaneous adiposity, maternal characteristics, and inflammation.

**Methods:**

Data of 520 undernourished children (250 of them had length-for-age *Z* score [LAZ] <−1 to −2 and 270 had LAZ score <−2) were collected from the Bangladesh Environmental Enteric Dysfunction study conducted in Dhaka, Bangladesh. Multivariable linear regression and generalized estimation equations (GEE) modeling techniques were used to measure the association.

**Results:**

At baseline, median RBP4 level was 19.9 mg/L (interquartile range [IQR]: 7.96), and at the end of the intervention, it was 20.6 mg/L (IQR: 9.06). Percentage changes in plasma RBP4 level were not significantly associated (*P* > .05) with the percentage changes in child’s height, weight, and subcutaneous adiposity. But maternal height (regression coefficient, β = −1.62, *P* = .002) and milk intake (β = −0.05, *P* = .01) were negatively and maternal weight was positively associated (β = 0.56, *P* = .03) with the changes in RBP4 levels. The GEE models revealed negative association of RBP4 levels with C-reactive protein (CRP; β = −0.14, *P* < .05) and α-1-acid glycoprotein (AGP; β = −0.03, *P* < .05).

**Conclusion:**

Children whose mothers were taller experienced less increase in plasma RBP4 level, and children whose mothers had a higher weight experienced more increase in the RBP4 level from baseline. We have also found that CRP and AGP levels and intake of whole milk were negatively associated with the plasma RBP4 level.

## Introduction

Retinol binding protein (RBP) is the principal carrier of vitamin A (retinol) in blood.^1^ It helps vertebrates to maintain a well-regulated retinol transportation system that can manage any fluctuations in vitamin A level.^2^ Retinol binding protein 4 (RBP4), a member of the RBP family, also functions as an adipocytokine.^3^ It has recently been reported to be associated with insulin resistance and several other components of the metabolic syndrome.^4^ Circulating RBP4 was found to have positive correlations with liver fat.^5^ Elevated plasma RBP4 levels might play a causative role in the development of systemic insulin resistance through immunity and inflammatory mechanisms.^6,7^ In GLUT-4 knockout mice, RBP4 expression was found to be upregulated in adipose tissue and a reduction in plasma RBP4 levels improved insulin action.^8^ Moreover, RBP4 is also regarded as an emerging marker of cardiometabolic risk factor.^9^

Multiple population-based studies have reported the impact of medical and nutritional interventions on plasma RBP4 levels. Plasma RBP4 was found to be reduced after intake of hypocaloric diets.^10^ Similar findings were reported from a study where morbidly obese humans reported a reduced plasma RBP4 level after losing their weight.^11^ Studies investigating the impact of bariatric surgery on circulating RBP4 reported a similar decreasing pattern.^10^ Obese children who went through a lifestyle intervention also showed a reduction in raised RBP4 levels.^12^ But all those studies were conducted among the overweight/obese adults and children and none of the studies explored the changes in plasma RBP4 level among the undernourished children after a nutrition intervention.

Undernutrition is a global public health concern. Worldwide, a total of 149 million preschool children are suffering from stunting.^13^ Nutrition scientists are directing different dietary interventions to eradicate undernutrition, in all its forms. A randomized controlled trial, conducted in Ecuador, delivered 1 egg per day for a 6-month period.^14^ The intervention increased length-for-age *Z* score (LAZ) by 0.63 and weight-for-age *Z* score by 0.61. In the Bangladesh Environmental Enteric Dysfunction (BEED) study, we supplemented the usual home diet of the mild, moderate, and severely stunted children with 1 boiled egg and 150 mL of whole milk for 90 feeding days and 1 sachet of multiple micronutrient powder daily for 60 feeding days.^15^ We found that the supplementation created a change of +0.27 (95% CI: 0.18-0.35; *P* < .05) in moderate/severe stunted (LAZ <−2) children and +0.19 (95% CI: 0.12-0.27; *P* < .05) in children with stunting (LAZ: −1 to −2).^16^ Although the outcome of interest of the study was improvement in linear growth, the changes in fatty mass and its associated changes in the markers of metabolic risk were also an arena to explore. Measuring the changes of plasma RBP4 level after the nutrition intervention is a way to predict any metabolic changes associated with the delivered intervention. Therefore, we investigated the changes in plasma RBP4 level among 12- to 18-month-old undernourished children after the above-mentioned nutrition intervention and measured its association with anthropometric status, subcutaneous adiposity, socioeconomic status, and maternal characteristics.

Undernutrition is closely associated with infection and inflammation. Systemic and gut inflammation is metabolically expensive and can result in adverse growth outcomes.^17^ Plasma RBP4, which is regarded as a negative acute phase reactant, is also frequently found to be reduced in settings of inflammation or infection.^12^ Hence, the aim of the current analysis also includes measuring the association of plasma RBP4 with the markers of systemic and gut inflammation.

## Methods

### Participants and Ethics

**Figure 1.**
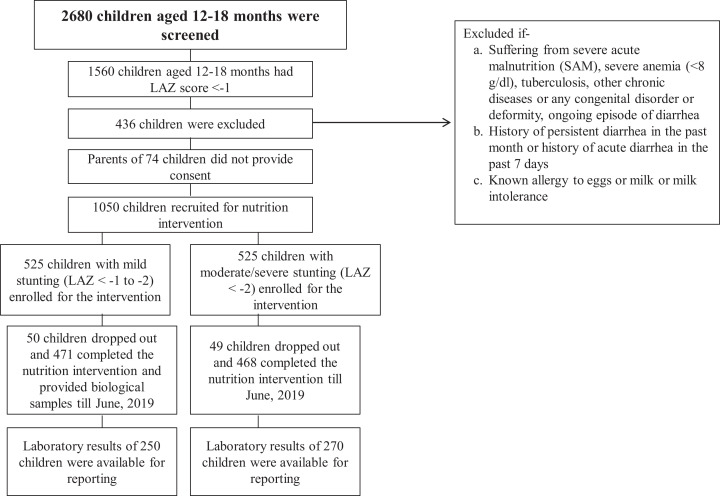
Participant flowchart showing the inclusion of participants in this study (period: July 2016-June 2019).

The BEED study (ClinicalTrials gov ID: NCT02812615) is a community-based intervention study which has been conducted in Bauniabadh slum area of Dhaka, Bangladesh. The study recruited undernourished children who were suffering from mild stunting (LAZ score <−1 to −2) or moderate/severe stunting (LAZ score <−2). Data from a total of 520 (250 mild stunting and 270 moderate/severe stunting) children, enrolled between July 2016 and June 2019, were used for this particular analysis. The enrollment scheme is shown as a flowchart in Figure 1. The study protocol was approved by the institutional review board of icddr, b. A trained field research assistant explained the study in detail, answered any questions from the parent(s), and invited the parent(s) to enroll the child in the study. Informed written consent was obtained from the mother or primary caregiver of all children. Incentives were offered to the mothers/primary caregivers of the children to compensate for their wage loss due to visiting the study office for facilitating the data collection. The detailed methodology of the study has been published elsewhere.^15^

### Data and Variable Description

Data on household socioeconomic status was collected from the mother/primary caregiver of the participants. Data collectors used pretested questionnaires and documented the information. WAMI index (Water, sanitation, hygiene, Asset, Maternal education and Income index; ranging from 0 to 1)—a socioeconomic status index which includes access to improved water and sanitation, 8 selected assets, maternal education, and household income was used as a representative of socioeconomic status of the households.^18^ A higher WAMI index indicates a better socioeconomic status. Morbidity data were collected daily for the duration of intervention. Diarrhea was defined as passage of ≥3 loose stools in 24 hours, and fever was defined as an axillary temperature of >99° F (37.2 °C) by a mercury thermometer.

Anthropometric measurements were conducted following an established and validated protocol.^19^ Children were weighed with very minimum clothing using a Seca digital scale (model no.727, 10 g precision), and a Seca infantometer (model no.416) was used to measure the recumbent length to the nearest 1 mm. The LAZ for each child was determined using the World Health Organization 2006 child growth standards.^20^ Harpenden skin fold calipers were used to measure the triceps skinfold (TSF) thicknesses. Standard measuring tapes were used to measure the mid upper arm circumference (MUAC). Seca stable stadiometer for mobile height measurement (model no.217, Hamburg, Germany, 0.1 cm precision) and Tanita step-on type weighing scales were used to measure the height and weight of the mothers, respectively. Regular quality control sessions were organized to ensure the reliability of the measurement. We regularly measured the Intraclass correlation coefficient (ICC) values to check the reliability of the measurers and found the ICC values above 0.9 in all the cases. Measuring equipments were also calibrated daily to maintain the validity of the measurements. Intake of egg and milk and related compliance data for the nutritional intervention were collected and recorded daily throughout the intervention period.

Upper arm fatty and fat-free mass areas were estimated following the mathematical formula proposed by Rolland-Cachera et al.^21^ The formula counts the upper arm area as a rectangle shaped unrolled fat rim whose length = upper arm circumference (C) and width = TSF thickness (TS)/2. Here, MUAC indicates the whole length of the total arm area or C, TSF thickness indicates total width of TS and total upper arm area (TUA) = c^2^/4π. Hence, the upper arm fat area (UFA) estimate = C × (TS/2), and upper arm muscle area estimate = TUA − UFA. The formula was validated using the magnetic resonance imaging technology.

All biological samples were collected following standard operative procedures which were prepared and validated for the study. A total of 5 mL venous blood and 2 g of stool samples were collected from each of the participants before and after the nutrition intervention. Blood samples were centrifuged at 4000 rotations per minute for 10 minutes to separate the plasma. Aliquots of stool and plasma were immediately frozen at −80 °C. Plasma RBP4 was measured using a commercially available enzyme-linked immunosorbent assay (ELISA) kit (R&D Systems Inc). A face mask was used during the laboratory test to protect the kit reagents from contamination as RBP4 is also found in saliva. Plasma samples were analyzed for C-reactive protein (CRP; Immundiagnostik), α-1-acid glycoprotein (AGP; Alpco), and ferritin (ORGENTEC Diagnostika GmbH) by using commercial ELISA kits. Stool samples were analyzed for neopterin (GenWay), myeloperoxidase (Alpco), and α-1-antitrypsin (A1AT; Biovendor) using commercially available ELISA according to kit manuals. Concentrations of each of the biomarkers were calculated against standards provided by the manufacturers.

### Statistical Analysis

Data analysis was conducted in R version 3.6.0 using the “dplyr,” “ggquickeda,” “ggplot2,” and “gee” packages. Exploratory data analysis was done to explore the socioeconomic, anthropometric, and morbidity status of the participants at baseline. Variables showing normal distribution are presented as mean and standard deviations (SDs), whereas variables with skewed distribution are presented as median and corresponding interquartile ranges (IQRs). Categorical variables are presented as frequency and percentages. Student *t* test and Mann-Whitney *U* test were used to detect the differences in the continuous variables, while chi-square test was used for comparing the categorical variables of the mildly stunted and moderate/severely stunted participants. Percentage changes of different anthropometric indices and plasma RBP4 level were calculated by deducting the after intervention values from the before intervention values, dividing the outcome by the before intervention values and multiplying by 100.

Multivariable linear regression analysis was done to measure the association of percentage change of plasma RBP4 level from baseline with subcutaneous adiposity, socioeconomic status, and maternal characteristics. After estimating the regression model, we also performed regression diagnostics to check for the assumptions of multicollinearity, homoscedasticity, and normality of residuals of the developed model using variance inflation factor (VIF), residuals versus fitted plot (rvf plot), and quantile–quantile (Q-Q) plot, respectively. We have found that all the VIF values were less than 2, indicating the absence of multicollinearity. The rvf plot showed that the heteroscedastic errors were not vividly present. The Q-Q plot indicates that the residuals were normally distributed. The results of the postestimation analyses reveal that the model estimated the coefficient accurately. All these results can be found in Supplementary File 1.

The association between plasma RBP4 level and the markers of inflammation at 2 time points (before and after the nutrition intervention) was examined longitudinally using population specific generalized estimation equations (GEE) modeling technique.^22^ The GEE method models the repeated responses collected from the same participants over time and produces efficient and unbiased regression parameters. For the reported analysis, the family was “Gaussian” and the link function was “Identity.” Quasi-likelihood information criterion (QIC) was used to select the correct covariance structure for building the GEE model, and the multivariable model with unstructured covariance matrix produced the smallest QIC value. Hence, we report the results of the multivariable model that was constructed using unstructured covariance matrix with robust variance estimates. We determined the strength of association by estimating the regression coefficients and their 95% CIs. In all analyses, a probability value <.05 was defined as the cutoff for statistical significance.

## Results

### Participant Characteristics

Table 1 describes the sociodemographic, maternal, and anthropometric characteristics of the participants. Nearly 50% of the participants were male. Mildly stunted children had a higher maternal height, weight, and family income than their counterparts. They also had a higher amount of fat and muscle mass in their upper arm than the moderate/severely stunted children. During the intervention period, children had fever and diarrhea on an average of 4 and 3 days, respectively.

**Table 1.** Sociodemographic, Anthropometric, and Morbidity Status of the Cohort.

Variables	Types of stunting			*P* value
	All (n = 520)	Mild stunting (n = 250)	Moderate/severe stunting (n = 270)	
Sociodemographic and maternal characteristics				
Sex (male), n (%)	252 (48.5)	113 (45.2)	139 (51.5)	.15
Age (days), mean (SD)	442 (65.7)	434 (65.0)	450 (65.5)	<.05
Mother’s height (cm), mean (SD)	149 (5.28)	151 (4.97)	148 (5.34)	<.05
Mother’s weight (kg), mean (SD)	47.8 (10.5)	52.0 (10.4)	49.9 (10.6)	<.05
Household income (USD), median (IQR)	172 (117)	179 (121)	163 (118)	<.05
Anthropometric indices at baseline				
Weight (kg), mean (SD)	8.11 (0.91)	8.47 (0.85)	7.78 (0.84)	<.05
Length (cm), mean (SD)	72.0 (2.9)	73.3 (2.41)	70.8 (2.74)	<.05
MUAC (cm), mean (SD)	13.7 (0.9)	13.9 (0.9)	13.5 (0.85)	<.05
Head circumference (cm), mean (SD)	43.9 (1.41)	44.2 (1.25)	43.7 (1.51)	<.05
Triceps skinfold thickness (mm), mean (SD)	6.79 (1.15)	6.90 (1.19)	6.69 (1.10)	<.05
Length-for-age *Z* score, mean (SD)	−2.18 (0.78)	−1.55 (0.29)	−2.77 (0.62)	<.05
Weight-for-age *Z* score, mean (SD)	−1.71 (0.87)	−1.25 (0.7)	−2.15 (0.8)	<.05
Weight-for-length *Z* score, mean (SD)	−0.882 (0.9)	−0.710 (0.9)	−1.04 (0.9)	<.05
Triceps skinfold-for-age *Z* score, mean (SD)	0.833 (0.85)	0.764 (0.9)	0.897 (0.83)	<.05
Upper arm fat area estimates (mm), mean (SD)	46.8 (9.96)	48.4 (10.5)	45.3 (9.22)	<.05
Upper arm muscle area estimates (mm), mean (SD)	1450 (191)	1500 (191)	1410 (180)	<.05
Morbidity days (during the intervention period)				
Fever, mean (SD)	4.15 (3.99)	3.77 (3.51)	4.50 (4.36)	.19
Diarrhea, mean (SD)	2.90 (3.61)	3.04 (3.63)	2.77 (3.60)	.22

Abbreviations: IQR, interquartile range; MUAC, mid upper arm circumference; SD, standard deviation.

### Changes in Different Anthropometric Indices After Nutrition Intervention

On average, everyday a child consumed 55 g of boiled egg and 149 mL of milk. Each micronutrient sachet contained 12.5 mg iron, 5 mg zinc, 300 µg vitamin A, 150 µg folic acid, and 50 mg of vitamin C (Monimix). After taking the intervention, overall, children gained more fat in their upper arm area than the muscle mass (5.90 vs 4.09 mm). Children from the mild stunting group gained more fat (mean ± SD) in the upper arm area (6.05 ± 21.1 mm) than the moderate/severe stunting group (5.79 ± 19.7 mm), whereas children with moderate/severe stunting gained more muscle mass (4.42 ± 9.57 mm) than the mildly stunted group (3.98 ± 8.93 mm). But the differences between the groups were not statistically significant (*P* > .05; Table 2).

**Table 2.** Percentage Changes in Different Anthropometric Indices From Baseline After Nutrition Intervention.

Variables	Types of stunting			*P* value^a^
	All (n = 520)	Mild stunting (n = 250)	Moderate/severe stunting (n = 270)	
Weight, median (IQR)	7.62 (6.48)	7.74 (6.30)	7.52 (6.75)	.79
Length, median (IQR)	4.96 (1.64)	4.95 (1.66)	4.96 (1.64)	.94
MUAC, median (IQR)	2.11 (4.48)	2.07 (4.23)	2.17 (4.60)	.52
Head circumference, median (IQR)	1.17 (0.94)	1.16 (1.13)	1.18 (0.92)	.39
Triceps skinfold thickness, median (IQR)	3.12 (18.6)	4.12 (18.4)	3.03 (18.9)	.36
Upper arm fat area estimates (mm), mean (SD)	5.90 (20.6)	6.05 (21.1)	5.79 (19.7)	.51
Upper arm muscle area estimates (mm), mean (SD)	4.09 (9.04)	3.98 (8.93)	4.42 (9.57)	.49

Abbreviations: IQR, interquartile range; MUAC, mid upper arm circumference; SD, standard deviation

^a^ Mild versus moderate/severe stunting; Mann-Whitney *U* test.

### Changes in Plasma RBP4 Level After the Nutrition Intervention and Its Association With Anthropometric Status, Subcutaneous Adiposity, Socioeconomic Status, and Maternal Characteristics

**Figure 2.**
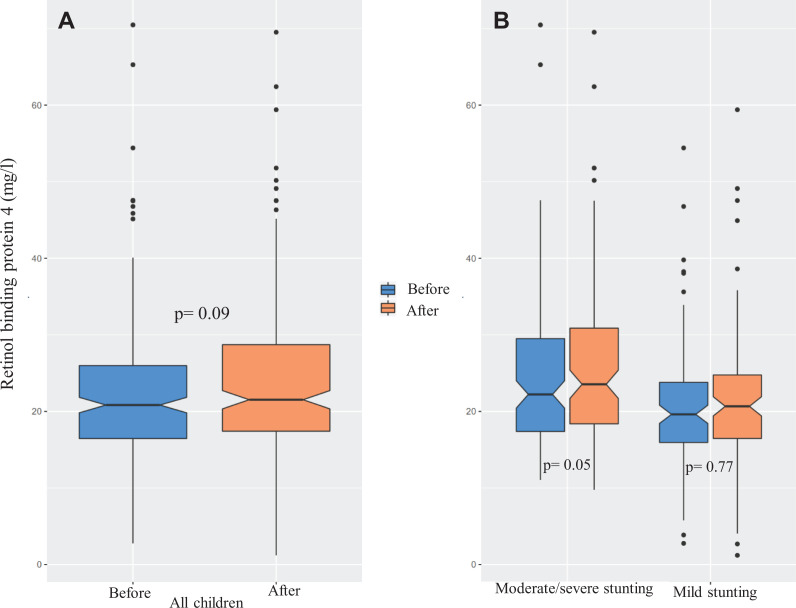
Levels of plasma retinol binding protein 4 (RBP4) before and after the nutrition intervention. Here panel A denotes the before–after changes in RBP4 level in all children and panel B presents the cohort specific before–after changes.

At baseline, median RBP4 level was 19.9 mg/L (IQR 7.96 mg/L), and at the end of the intervention, it was 20.6 mg/L (IQR: 9.06 mg/L). Overall, the children gained a median of 1.73% (IQR: 45.0 mg/L) increase in plasma RBP4 level from baseline. At baseline, moderate/severe stunting group had a higher plasma RBP4 level (median [IQR]: 21.0 [9.25] mg/L) than the mildly stunted group (median [IQR]: 18.9 [7.59] mg/L). And at the end line, the values were (median [IQR]: 22.1 [10.3] mg/L) and (median [IQR]: 19.6 [8.11] mg/L), respectively (Figure 2).

Table 3 presents the association of percentage changes in plasma RBP4 level with anthropometric status, subcutaneous adiposity, socioeconomic status, and maternal characteristics. The multivariable linear regression model showed significant (*P* value <.05) association of the outcome variable with maternal height, maternal weight, and milk intake. Maternal height (regression coefficient β: −1.65; 95% CI: −2.67 to −0.62; *P* < .05) and milk intake (β: −0.05; 95% CI: −0.08 to 0.01; *P* = .01) were negatively associated with the percentage change of plasma RBP4 level, whereas maternal weight (β: 0.56; 95% CI: 0.05-1.07; *P* = .03) was positively associated with it. Variables indicating the changes in anthropometry, socioeconomic status, and morbidity did not show any statistically significant (*P* > .05) association with the outcome variable in the fully adjusted model.

**Table 3.** Association of Percentage Changes in Plasma RBP4 level With Anthropometric Status, Subcutaneous Adiposity, Socioeconomic Status, and Maternal Characteristics: Results of Multivariable Linear Regression.

Variables	Unadjusted				Adjusted			
	β	95% CI		*P* value	β	95% CI		*P* value
		Lower	Upper			Lower	Upper	
Percentage change in weight	1.04	0.02	2.06	.05	−0.01	−1.30	1.28	.99
Percentage change in height	3.02	−1.14	7.18	.15	2.30	−2.13	6.73	.31
Percentage change in UFA	0.41	0.10	0.71	.01	0.35	−0.03	0.73	.07
Percentage change in UMA	0.61	−0.05	1.26	.07	0.22	−0.58	1.01	.59
WAMI index^a^	3.38	−33.01	39.76	.86	1.37	−36.42	39.15	.94
Gender	5.82	−4.19	15.83	.25	4.32	−5.73	14.36	.40
Mother’s height	−1.22	−2.16	−0.27	.01	−1.65	2.67	−0.62	.00
Egg intake	0.00	−0.02	0.02	.75	0.02	−0.01	0.04	.17
Milk intake	−0.02	−0.05	0.00	.10	−0.05	0.08	−0.01	.01
Age in days	−0.02	−0.10	0.06	.61	−0.02	−0.10	0.06	.66
Stunting group	1.58	−8.44	11.61	.76	1.79	−8.75	12.32	.74
Mother’s weight	0.27	−0.20	0.74	.25	0.56	0.05	1.07	.03
Fever days	−0.62	−1.87	0.64	.33	−0.48	−1.82	0.86	.48
Diarrhea days	−0.58	−1.97	0.81	.41	−0.21	−1.63	1.21	.77

Abbreviations: RBP4, retinol binding protein 4; UFA, upper arm fat area; UMA, upper arm muscle area.

^a^ WAMI index (Water, sanitation, hygiene, Asset, Maternal education and Income index; ranging from 0 to 1)—a socioeconomic status index which includes access to improved water and sanitation, eight selected assets, maternal education, and household income was used as a representative of socioeconomic status of the households.

### Levels of Different Biomarkers of Inflammation and Immune Response Before and After Nutrition Intervention

**Figure 3.**
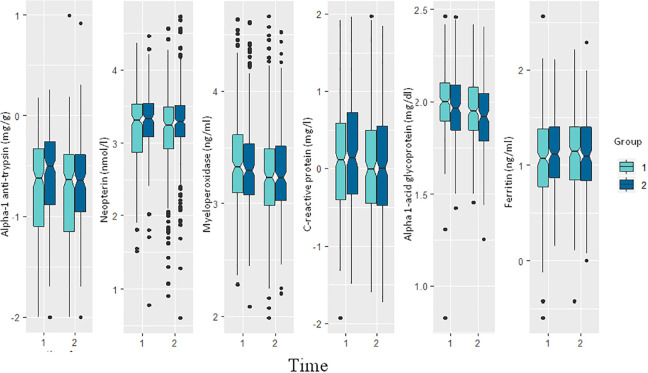
Levels of different biomarkers of systemic and gut inflammation before and after the nutrition intervention. Here time 1 denotes before intervention, time 2 denotes after intervention, group 1 denotes moderate/severely stunted group and group 2 denotes mildly stunted group.

Figure 3 presents the distribution of the markers of systemic and gut inflammation and immune response of mild and moderate/severely stunted children before and after the nutrition intervention. Except plasma ferritin, values of the other markers went down after the nutrition intervention. The pattern was same for both the cohorts.

### Association of Plasma RBP4 With the Markers of Inflammation and Immune Response

The multivariable GEE models revealed statistically significant (*P* < .05) negative association between CRP (β: −0.14; 95% CI: −0.15 to −0.13; *P* < 005), AGP (β: −0.03; 95% CI: −0.04 to 0.02; *P* < .05), and RBP4 levels (Table 4). We also found that the association of RBP4 with A1AT and ferritin was not statistically significant and the neopterin and myeloperoxidase had a near-zero association with the outcome variable, plasma RBP4 level.

**Table 4.** Association of the Biomarkers of Inflammation With the Plasma RBP4 Level: Results of GEE Modeling.

Variables	Unadjusted				Adjusted^a^			
	β	95% CI		*P* value	β	95% CI		*P* value
		Lower	Upper			Lower	Upper	
Alpha-1 anti-trypsin	−0.22	−0.79	0.35	0.46	−0.05	−0.64	0.54	0.87
Neopterin	0.00	0.00	0.00	0.07	0.00	0.00	0.00	0.02
Myeloperoxidase	0.00	0.00	0.00	0.01	0.00	0.00	0.00	0.01
C-reactive protein	−0.15	−0.22	−0.08	0.00	−0.14	−0.15	−0.13	0.00
α-1-acid glycoprotein	−0.03	−0.04	−0.01	0.00	−0.03	−0.04	−0.02	0.00
Ferritin	0.01	−0.01	0.03	0.00	0.01	−0.01	0.03	0.32

Abbreviations: GEE, generalized estimation equation; RBP4, retinol binding protein 4; WAMI, Water, sanitation, hygiene, Asset, Maternal education and Income index.

^a^ All the models are adjusted for age at enrollment, sex, malnutrition status at baseline, and WAMI index.

## Discussion

The analyses revealed that, in addition to improving the height and weight of the children, a nutrition intervention comprising egg and whole milk can result in accumulation of more subcutaneous fat than muscle mass in the upper arm area. But the intervention did not increase the risk of alteration of metabolic profile as the levels of RBP4 in plasma did not change significantly after the nutrition intervention. Moreover, milk intake was found to be negatively associated with the percentage change of plasma RBP4 level, indicating a protective change against the development of cardiometabolic risk. Whole milk is one of the top sources of saturated fat. Although different dietary guidelines recommended reducing intake of saturated fat for a better cardiometabolic health,^23^ the evidence emanating from recent observational and intervention studies suggests that full-fat dairy products do not increase the risk of insulin sensitivity and cardiometabolic disease risk, rather exert potentially beneficial effects.^24-27^ These findings are similar to our study, but the downstream molecular pathways that might be responsible for this still remain unidentified. It has been hypothesized that the combined action of short chain fatty acids (SCFAs), protein, calcium, vitamin D, and probiotics present in milk and dairy products might be responsible for exerting the beneficial effects.^23^ Short chain fatty acids can independently affect lipid, glucose, and cholesterol metabolism in various tissues.^28,29^ Moreover, SCFAs and probiotics have the potential to modulate the effect of dairy-based foods on cardiometabolic function by interacting with the microbial community of the host gut.^30^

We have found that children, whose mothers were taller, experienced less increase in plasma RBP4 level from baseline than their counterparts. On the other hand, children, whose mother had a higher weight, experienced more increase in the RBP4 level. There is a paucity of data for undernourished infants that might echo our findings. But it has been shown that among the older children (aged 6-11 years), maternal obesity exerted a 2-fold risk of developing metabolic syndrome.^31^ The “Developmental Origins of Adult Health and Disease” hypothesis states that children facing any unfavorable conditions during the early postnatal periods might be at a greater risk of developing adulthood diseases.^32^ However, the vectors that might potentially modify the mechanistic underpinnings of the phenomenon are yet to be explored and genetic and epigenetic factors are the potential candidates for that exploration. It has recently been hypothesized that maternal diet, which is directly related to the maternal obesity, might alter the methylation status of insulin-like growth factors and related genes in infants.^33^ Although more studies are needed to prove or refute the hypothesis, scientists have shown that maternal overnutrition could program the metabolic phenotypes of the offspring through sperm tRNA-derived small RNAs.^34^ Overall, this is an interesting area where we have tried to shed the light, and molecular level studies are now required to explore the exact relation.

In addition to liver, adipose tissue also synthesizes and secretes RBP4, and the expression is higher in visceral fat compared to subcutaneous fat.^35,36^ Accordingly, the circulating levels of RBP4 show stronger correlation to the quantity of visceral fat than the subcutaneous fat.^36^ Hence, changes in subcutaneous fat are unlikely to show any strong association with the RBP4 level. Multivariable linear regression done in our study reveals the same as we have found that changes in fat and fat-free mass of upper arm area were not significantly associated with the changes in plasma RBP4 level.

From the descriptive analysis, we have found that the levels of RBP4 increased after the nutrition intervention, whereas the levels of most of the positive acute phase reactants (those which increase in response to inflammation) decreased. These findings indicate that intake of animal protein based foods might impact the inflammation process by modifying the actions of positive and negative acute phase proteins. A Mediterranean diet that contains high amount of monounsaturated and ω-3 polyunsaturated fatty acid has shown similar anti-inflammatory effects.^37^ Cow’s milk and egg yolk are known to be the rich sources of fatty acids.^38^ Hence, regular intake of whole milk and chicken eggs might play important roles in combating inflammation as our study showed a declining trend in the levels of inflammatory biomarkers after the nutritional intervention. The GEE analyses revealed that plasma RBP4 level had a statistically significant negative association with CRP and AGP. Very few studies have explored the role of RBP4 in systemic inflammation, more so in the absence of obesity and related metabolic alterations. A review paper published on the role of RBP4 in inflammation revealed mixed results. Of 21 papers reviewed, only 2 studies reported negative relations and 4 studies reported positive association. But the rest of the studies (15) could not find any relation between RBP4 and CRP.^39,40^ But being a negative acute phase reactant, the negative association of circulatory levels of RBP4 with CRP and AGP implies biological plausibility. The association of RBP4 with the rest of the inflammatory markers showed statistically insignificant relationships with negligible effect sizes.

This is the first study that has compared the before–after changes of plasma RBP4 level among the undernourished children and measured its association with the body composition, biomarkers of inflammation, and maternal nutritional status. But we could not compare those changes with age and sex matched healthy cohort of control children. Moreover, we did not measure visceral adiposity and serum levels of vitamin A. These are the limitations of our study.

## Conclusions

In summary, our study reports that plasma RBP4 level, a marker of metabolic risk and insulin resistance, is positively associated with mother’s weight but negatively with mother’s height. Intake of whole milk is negatively associated with the percentage change of plasma RBP4 level after the nutrition intervention. We have also found that the markers of systemic inflammation were negatively associated with plasma RBP4 level.

## Supplemental Material

Supplemental Material, Supplementary_file - Changes in Retinol Binding Protein 4 Level in Undernourished Children After a Nutrition Intervention Are Positively Associated With Mother’s Weight but Negatively With Mother’s Height, Intake of Whole Milk, and Markers of Systemic Inflammation: Results From a Community-Based Intervention Study Click here for additional data file. Supplemental Material, Supplementary_file for Changes in Retinol Binding Protein 4 Level in Undernourished Children After a Nutrition Intervention Are Positively Associated With Mother’s Weight but Negatively With Mother’s Height, Intake of Whole Milk, and Markers of Systemic Inflammation: Results From a Community-Based Intervention Study by Subhasish Das, Md Amran Gazi, Md Mehedi Hasan, Shah Mohammad Fahim, Md Ashraful Alam, Md Shabab Hossain, Mustafa Mahfuz and Tahmeed Ahmed in Food and Nutrition Bulletin
