# Supplementary material for: Changes in Retinol Binding Protein 4 Level in Undernourished Children After a Nutrition Intervention Are Positively Associated With Mother’s Weight but Negatively With Mother’s Height, Intake of Whole Milk, and Markers of Systemic Inflammation: Results From a Community-Based Intervention Study
Source: Food Nutr Bull. 2020 Nov 23;42(1):23–35. doi: 10.1177/0379572120973908 (PMC8060731; doi:10.1177/0379572120973908)

Results of the post-estimation analyses of multivariable linear regression

1. Variance inflation factor (VIF) values of the explanatory variables

Variables	VIF values
Percent change in weight	1.62
Percent change in height	1.16
Percent change in UFA	1.58
Percent change in UMA	1.50
WAMI index	1.11
Gender	1.03
Mother's height	1.20
Egg intake	1.74
Milk intake	1.72
Age in days	1.14
Stunting group	1.14
Mother's weight	1.20
Fever days	1.16
Diarrhea days	1.08

2. Residuals vs. fitted plot (rvf plot)

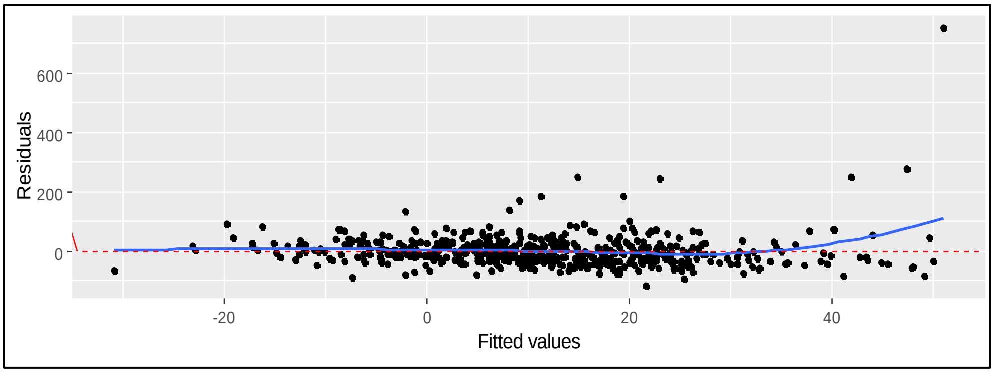

3. Quantile-Quantile (Q-Q) plot

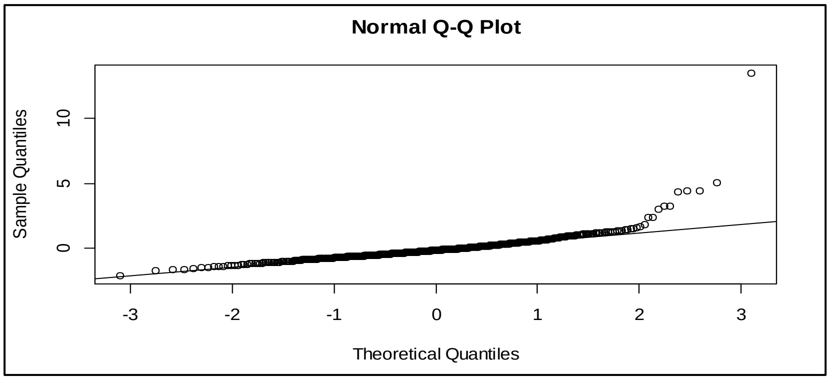

Supplement: Supplemental Material, Supplementary_file - Changes in Retinol Binding Protein 4 Level in Undernourished Children After a Nutrition Intervention Are Positively Associated With Mother’s Weight but Negatively With Mother’s Height, Intake of Whole Milk, and Markers of Systemic Inflammation: Results Fro [file Supplementary_file.pdf]
